# 

**Published:** 1878-02

**Authors:** 


					﻿Table of Contents.
-*OIHOINAL 4'()MMLN1(’ATI<)NS.
Is 5J(»i>ki!X Knication Kxertinir an Evil Infitience upon the Eve-
sight of ^’oiir ('hihlren ? lit/ [l. IE. Cfdlnnm. M.h., Alhmln.
(Jeoryiu........................................................... *>11
'I’liRKE P'amiijes Poisoned hy Eating lee Cream, at Macon, (Ja.
'I he Source of the PoisOn, etc. />// Wllliniii ,1. (Ifeeuf,
Mm-ou, (jta.....................................................
Oi’ii M IN Hernia. Hh A. fAnirt'i-t. M.D., (fxluril, (in......... (i7.')
PEPO RTS OK SOCIE'I'IES.
.Olanta Academy of .Metlicine...................................... liii
SELECTIONS.
Vihurniiiii Prunifolinm as a Cterine Sislative..................... IlSl
Nitrate of Pitocarpia.............................................. hs.)
Salicylic Aciil and Salicylati* of Soda in tin* '1 r(*atm(*nt ot Acute
Pheninatism..........................................................
'flu* P«.*sponsihility of the Prof«*ssion in the Production of Opium
Inebriety............................................................
EDITORIAL.
Hospitals.....................................................     hit!)
Medical Association of	Oeorgia..................................... 7(K>
In .Memoriam.......................................................   RR
I'Mitorial Correspondence......................................... '•).>
				

